# Structural variation during dog domestication: insights from gray wolf and dhole genomes

**DOI:** 10.1093/nsr/nwy076

**Published:** 2018-07-19

**Authors:** Guo-Dong Wang, Xiu-Juan Shao, Bing Bai, Junlong Wang, Xiaobo Wang, Xue Cao, Yan-Hu Liu, Xuan Wang, Ting-Ting Yin, Shao-Jie Zhang, Yan Lu, Zechong Wang, Lu Wang, Wenming Zhao, Bing Zhang, Jue Ruan, Ya-Ping Zhang

**Affiliations:** 1 State Key Laboratory of Genetic Resources and Evolution, Kunming Institute of Zoology, Chinese Academy of Sciences, Kunming 650223, China; 2 Center for Excellence in Animal Evolution and Genetics, Chinese Academy of Sciences, Kunming 650223, China; 3 Agricultural Genomics Institute, Chinese Academy of Agricultural Sciences, Shenzhen 518120, China; 4 Medical Faculty, Kunming University of Science and Technology, Kunming 650504, China; 5 Department of Pediatrics, the First People's Hospital of Yunnan Province, Kunming 650032, China; 6 College of Pharmacology, Soochow University, Suzhou 215123, China; 7 Key Laboratory of Animal Models and Human Disease Mechanisms of the Chinese Academy of Sciences and Yunnan Province, Kunming Institute of Zoology, Chinese Academy of Sciences, Kunming 650223, China; 8 Department of Laboratory Animal Science, Kunming Medical University, Kunming 650500, China; 9 Laboratory for Conservation and Utilization of Bio-Resources and Key Laboratory for Microbial Resources of the Ministry of Education, Yunnan University, Kunming 650091, China; 10 Kunming College of Life Science, University of Chinese Academy of Sciences, Kunming 650204, China; 11 Beijing Zoo, Beijing 100044, China; 12 Core Genomic Facility, Beijing Institute of Genomics, Chinese Academy of Sciences, Beijing 100101, China

**Keywords:** dog domestication, genome assembly, structural variation, gray wolf, dhole

## Abstract

Several processes like phenotypic evolution, disease susceptibility and environmental adaptations, which fashion the domestication of animals, are largely attributable to structural variations (SVs) in the genome. Here, we present high-quality draft genomes of the gray wolf (*Canis lupus*) and dhole (*Cuon alpinus*) with scaffold N50 of 6.04 Mb and 3.96 Mb, respectively. Sequence alignment comprising genomes of three canid species reveals SVs specific to the dog, particularly 16 315 insertions, 2565 deletions, 443 repeats, 16 inversions and 15 translocations. Functional annotation of the dog SVs associated with genes indicates their enrichments in energy metabolisms, neurological processes and immune systems. Interestingly, we identify and verify at population level an insertion fully covering a copy of the *AKR1B1* (Aldo-Keto Reductase Family 1 Member B) transcript. Transcriptome analysis reveals a high level of expression of the new *AKR1B1* copy in the small intestine and liver, implying an increase in *de novo* fatty acid synthesis and antioxidant ability in dog compared to gray wolf, likely in response to dietary shifts during the agricultural revolution. For the first time, we report a comprehensive analysis of the evolutionary dynamics of SVs during the domestication step of dogs. Our findings demonstrate that retroposition can birth new genes to facilitate domestication, and affirm the importance of large-scale genomic variants in domestication studies.

## INTRODUCTION

Besides single-base and short-segment variants (≤ 50bp), genetic variation comprises larger chromosomal events such as large deletions, large insertions, inversions, duplications and translocations, all of which could be categorized as structural variations (SVs) [1,2]. SVs have pronounced genomic impacts, including directly affecting gene dosage, indirectly altering gene expression through the position effect, unmasking recessive alleles or regulatory polymorphisms, losing regulatory elements and affecting the evolution of new genes [3–6]. Being an important source of genetic variation, SVs have prominent roles in phenotypic evolution, disease susceptibility and environmental adaptation, which are critical processes in the domestication of animals [7,8]. For instance, a several-fold increase of *AMY2B* in domestic dog enabled its adaptation to a starch-rich diet and the establishment of the close human–dog bond [9]. A duplication of *ASIP* in sheep leads to white and black pigmentation [10]. A copy number gain of the genomic segment containing the *KIT* gene causes the dominant white-coat phenotype in different European pig breeds [11,12]. Copy number changes of the *TSEG2*, *AKR1C3* and *IZUMO1* genes, which relate to spermatogenic cell development and fertility, enhance the reproductive ability of yaks [7].

As the first animal to be domesticated, dogs have participated in many aspects of human lives, making their domestication of great interest and significance to human society [13–16]. Furthermore, their diverse phenotypic variation makes them an ideal model to study the effects of domestication and artificial selection [17–19]. With the current advances in resequencing approaches, more light continues to emerge on subjects of significant scientific interest such as the demographic history, origins, admixture and environmental adaptation of dogs [20–23]. However, SVs and their involvement in dog domestication are still poorly understood. One reason is the unequal attention paid to small- and large-scale variants compared to single-nucleotide polymorphisms (SNPs). SNPs and indels are so far the most widely used genetic markers in the investigation of dog evolution [17,24,25]. The voids in the study of dog genome SVs could be at the expense of important genetic signals that could further clarify the domestication of dogs. The other drawback is the lack of genome assemblies of dogs' wild ancestors and outgroups, both of which are essential in identifying ancestral and lineage-specific SVs, and could facilitate the characterization of vital events in the evolutionary histories of certain species [26,27]. Reliance on the dog genome as a reference inevitably overlooks wolf-specific information, especially large chromosomal variations, and cannot decipher the evolutionary dynamics of SVs during domestication.

In order to advance our understanding of the domestication of dogs, we present the first annotated genome assembly of dog's wild ancestor, the gray wolf (*Canis lupus*), and the first *de novo* genome assembly of a dhole (*Cuon alpinus*) as outgroup. Through comparative analysis, we identify the key dog-specific transposable elements (TEs), gene family alterations and SVs, and analyze their contribution to phenotypic variations that characterize dog domestication.

## RESULTS

### Genome sequence, assembly and annotation

The gray wolf (*C*. *lupus*) sample was collected from Shandong province, China, and the specimen of dhole (*Cu*. *alpinus*) was sampled from Beijing Zoo, China. The dhole (*Cu*. *alpinus*) is a canid native to Central, South and Southeast Asia, which is genetically close to species within the genus *Canis* [28]. We constructed libraries of various insert sizes for sequencing by Ilumina HiSeq 2000, including four paired-end libraries and three mate-pair libraries of the dhole, and six paired-end libraries and five mate-pair libraries of the gray wolf. These libraries provide 145-fold and 81-fold base pair coverage of the gray wolf and dhole genomes, respectively (Supplementary Tables S1 and S2). Estimation of genome size based on k-mer depth distributions of raw sequencing reads are 2.41 Gb for the gray wolf and 2.63 Gb for the dhole, respectively (Supplementary Fig. S1).

The gray wolf genome assembly is 2.31 Gb with a scaffold N50 size of 6.04 Mb, while that of dhole is 2.33 Gb with a scaffold N50 size of 3.96Mb (Table 1). The GC content in the gray wolf and dhole genomes is 40.7 and 41.26%, respectively (Supplementary Fig. S2), which is similar to the GC content of the dog reference genome. The repeat content of the gray wolf, dhole and dog genomes is summarized in Supplementary Tables S3–S5. TEs account for 41.75% of the dog genome assembly, higher than the gray wolf genome (39.26%) and the dhole genome (38.51%). We built consensus sequences of all TEs of each genome separately by RepeatModeler to search for TE homology. These two annotation approaches were combined to account for all TEs in the three genomes (Supplementary Tables S6–S8). In the end, the proportion of TEs in the dog genome is still higher than those in the other two genomes (40.31, 39.13 and 38.51% in the dog, gray wolf and dhole genomes, respectively). The most divergent components of the repeated elements are Long Interspersed Nuclear Element/L1s (LINE/L1s) and satellites, both making up 86.1% of the differentiation between dog and gray wolf, and 83.2% between dog and dhole. A closer look into the distribution of the LINE/L1s in all three genomes shows that the percentages of L1_Canis1 and L1_Cf in the dog genome are significantly higher and almost twice those of the other two canine genomes (Table 2 and Supplementary Table S9). This pattern suggests that the L1_Canis1 and L1_Cf subfamilies might have been accumulated in the dog genome during domestication. We also assessed SINEC_Cf elements since they have undergone recent expansion in dog domestication [29,30]. Our results indicate that the dog genome contains 27.3 million SINEC_Cf elements, 1.16 times greater than in the wolf genome and 1.23 times greater than in the dhole genome (Table 2).

**Table 1.** Assembly statistics of wolf and dhole genomes, respectively.

	Wolf		Dhole	
	Contig	Scaffold	Contig	Scaffold
**Total Size**	2 259 426 957	2 313 148 660	2 288 150 950	2 329 418 464
**Total number**	103 755	44 203	68 931	29 680
**Average size**	21 777	52 330	33 195	78 484
**Median**	2997	282	6924	348
**Longest**	1 005 460	27 629 335	901 812	22 239 291
**N50**	83 801	6 037 699	113 743	3 955 117
**N90**	17 452	813 143	25 060	590 962

**Table 2.** The ratio of canid-specific LINEs and SINEs in the genomes of dhole, gray wolf and dog, respectively.

	Subclass	Dog	Wolf	Ratio (dog/wolf)	Dhole	Ratio (dog/dhole)
**LINE/L1**	L1_Canid	14 420 953	13 224 994	1.09	12 864 093	1.12
	L1_Canid2	3 902 975	3 709 460	1.05	3 645 582	1.07
	L1_Canis1	65 757 351	30 116 012	2.18	27 781 790	2.37
	L1_Canis2	5 041 715	7 270 039	0.69	6 577 792	0.77
	L1_Cf	13 740 230	6 842 615	2.01	6 121 615	2.24
**SINE**	SINEC_Cf	28 364 011	24 554 748	1.16	23 152 948	1.23

*De novo* predictions and homolog-based predictions were integrated to annotate the protein-coding genes in both genomes. We also used transcriptomic data from the liver, tongue, olfactory bulb and caudate nucleus for the annotation of the wolf genome. In summary, 20 045 and 20 797 high-confidence protein-coding genes were identified in the dhole and wolf genomes, respectively (Supplementary Tables S10 and S11). Of the 19 256 and 18 887 predicted genes in the dhole and wolf genomes, we successfully annotated > 96 and 90%, respectively, based on the functional protein databases (Supplementary Table S12). We used BUSCO to check the genome assembly and annotation [31]. The predicted proteins represent 95.8 and 91.6% matches with the set of 4104 BUSCO genes in dhole and wolf, respectively (Supplementary Table S13). Furthermore, Canidae EST sequences from the National Center for Biotechnology Information were separately mapped to protein-coding sequences of gray wolf, dhole and dog. The results show a similar ratio to EST mapping, indicating the high accuracy of gene predictions of both the gray wolf and dhole genomes (Supplementary Table S14). To estimate the completeness and accuracy of gene boundaries, 248 human core eukaryotic genes were aligned against peptide sequences of the three genomes separately. Similar ratios were obtained for gray wolf, dhole and dog, as shown in Supplementary Table S15, which verifies the robustness of the gene predictions in gray wolf and dhole.

**Figure 1.**
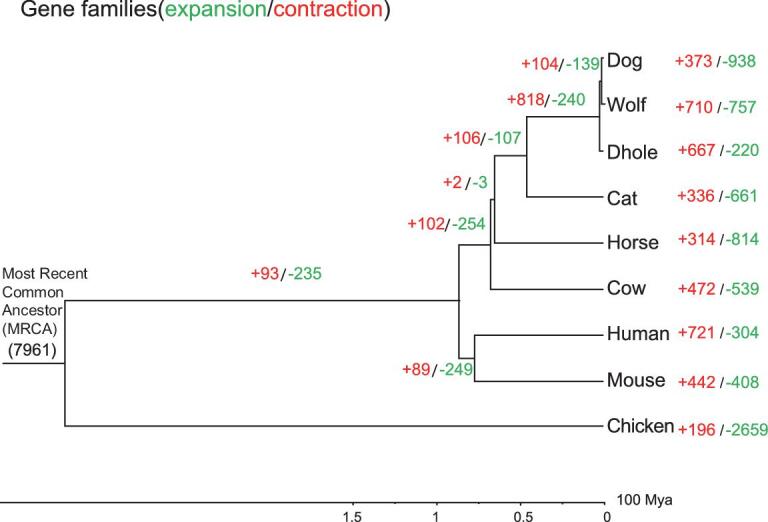
Construction of mammalian gene families. Red letters represent the number of expanded gene families and green letters represent the number of contracted gene families.

We detected 9754 gene families across the dog, dhole and gray wolf genomes. As shown in Fig. 1, there are more contracted than expanded gene families in the dog genome compared to the gray wolf and dhole common ancestors. Chi-square test comparison of observed and expected numbers of gene families shows significantly higher numbers of contracted than expanded gene families (*P*-value ≤ 2.2e-16). InterPro classification of expanded genes shows a significant enrichment (*P* < 0.01 by Fisher's exact test and *P* < 0.05 after false discovery rate correction) in the functional categories of energy/nutriment metabolism (207 genes), neurological processes (100 genes) and tumor/immune processes (167 genes).

### Whole-genome alignments of three Caninae genomes

**Figure 2.**
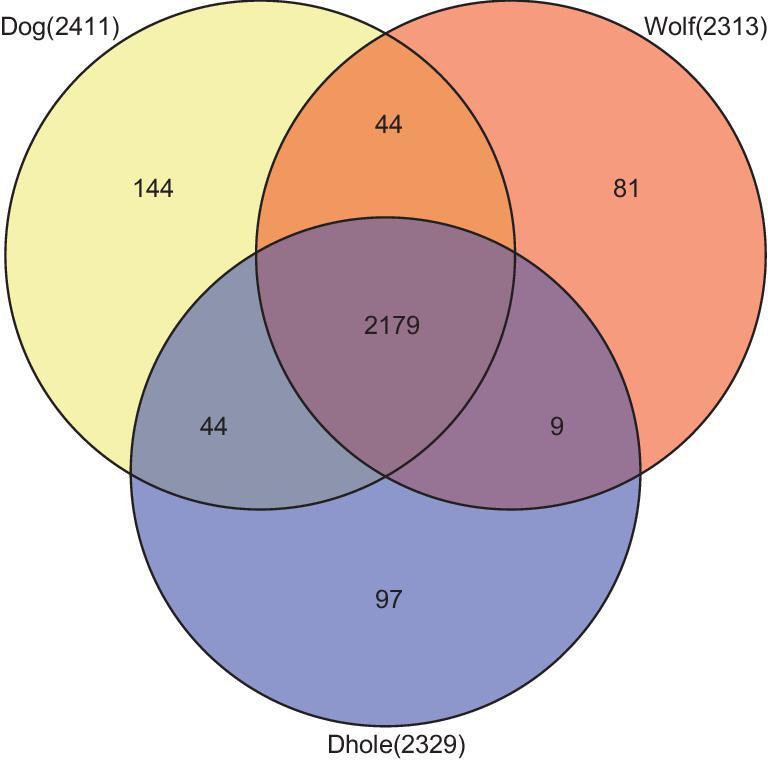
Lineage-specific sequence contents of dog, wolf and dhole genomes. The red, yellow and purple circles represent the gray wolf (2313 Mb), dog (2411 Mb) and dhole genomes (2329 Mb), respectively. The non-overlapping parts represent lineage-specific regions of each genome.

We performed whole-genome alignments to identify SVs among the three canine genomes. Firstly, we conducted pairwise whole-genome alignment among the dog, gray wolf and dhole genomes, and extracted orthologous alignment blocks. Subsequently, we linked the scaffolds of wolf and dhole to the dog reference genome to identify SVs related to dog domestication. The genome-scaled alignments reveal a conserved synteny of the canine genomes, with only a few interchromosome rearrangements (Fig. 2). Up to 97.33% of the gray wolf genome and 96.67% of the dhole genome could be placed onto the dog genome (Supplementary Table S16). In particular, 96–97% of the dog autosomes can be covered by the wolf and dhole scaffolds, while 90.0 and 86.1% of dog X chromosome can be covered by wolf and dhole scaffolds, respectively.

### Regions specific to the dog genome

**Figure 3.**
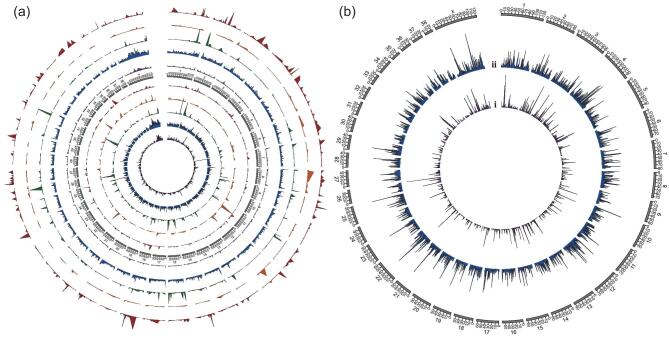
Structural variation in the dog genome. (a) Circle diagram showing SVs detected by the dog–dhole alignment (yellow) and the dog–wolf alignment (black). (b) SVs in the dog genome by identified by multiz alignment. Each ring from the inner ring outwards represents translocations, insertions, deletions, repeats and inversions, respectively.

Based on the multiple alignment result, we analyzed the shared and lineage-specific sequence content across dog, wolf and dhole, as shown in Fig. 3. The results show that there are 2179 Mb genomic regions that can be aligned in all three canine genomes, covering 91.5% of the dog genome. However, 143.9, 80.7 and 97.4 Mb genomic regions are specific to the dog, gray wolf and dhole genomes, respectively, showing that the dog genome contains more specific regions than the other canines. We identified 215 intact protein-coding genes (Supplementary Table S17) and 31 long intergenic non-coding transcripts (lincRNAs) (Supplementary Table S18) in the regions specific to the dog genome. Enrichment analysis shows that the dog-specific genes were significantly enriched in the olfactory transduction, ribosome, drug metabolisms, and starch and sucrose metabolism terms (Supplementary Table S19).

After combining the gene list with that of the expansion gene families, 38 genes remain unmatched. Olfactory transduction and immunoglobulin kappa chain V-II region gene families are the largest. Apart from these two big gene families, starch and sucrose metabolism, the immune system and neurological processes show strong prominence in our analysis. For instance, our approach identified *AMY2B*, which was reported by a previous study to be related to the digestion of dietary starch [9]. We also identified *IFNGR2* (Interferon Gamma Receptor 2) and *SIRPB1* (Signal-Regulatory Protein Beta 1), both located in dog-specific genomic regions. *IFNGR2* is subunit of the activated IFN-γ receptor complex [32], while *SIRPB1* is a member of the signal-regulatory-protein family, which belongs to the immunoglobulin superfamily [33]. Interestingly, a copy number polymorphism of *SIRPB1* is a candidate quantitative trait locus for impulsive-disinhibited personality [34]. Additionally, we identified *ATP5O* (ATP Synthase, H+ Transporting, Mitochondrial F1 Complex, O Subunit), which is related to neurodegenerative diseases associated with mitochondrial dysfunction [35].

### SVs during dog domestication

Based on whole-genome alignments, we explored the SVs between the dog and dhole, and dog and wolf, genomes (Table 3). We identified 115 937 insertions, 63 809 deletions, 4248 repeats, 1416 translocations and 463 inversions in the dog–dhole comparison (Supplementary Table S20). Meanwhile, there were 76 889 insertions, 28 482 deletions, 3921 repeats, 1618 translocations, and 368 inversions in the dog–wolf comparison (Supplementary Table S21), which are generally less than those identified in the dog–dhole alignment. It is evident that insertions and deletions are the dominant components, accounting for > 90% of SVs in both alignments. Subsequently, we analyzed the intersection of SVs with various classes of genic and intergenic functional elements. In both the dog–dhole and dog–wolf alignments, most of SVs fall into non-coding regions such as intergenic untranslated regions (UTRs), and intron locations, while few SVs intersect with coding regions. Taking deletions and insertions as examples, intergenic UTRs and intron locations contain ∼86 and ∼ 88% of the SVs in the dog–dhole and dog–wolf alignments, respectively, while coding sequence (CDS) and exon regions only account for ∼0.03 and ∼0.15% of total the SV number in the two respective alignments.

**Table 3.** A summary of the different types of SVs.

SV	Dog vs. dhole	Median size	Dog vs. wolf	Median size	Multiz	Median size
**Inversion**	463	213	368	196.5	15	335
**Repeat (from lastz overlap)**	4248	1785	3921	1874	443	1876
**Insertion**	115 937	207	76 889	201	16 315	213
**Deletion**	63 809	209	28 482	217	2565	168
**Translocation**	1416	273	1618	405	16	603

In order to find key variants for dog domestication and eliminate potential wolf- or dhole-specific noise, we detected SVs unique to dog through multiple alignments. As shown in Supplementary Table S22, dog-specific SVs include 15 inversions, 443 repeats, 16 315 insertions, 2565 deletions and 32 translocations. Of these, 0, 47, 89, 31 and 4 inversions, repeats, insertions, deletions and translocations, respectively, overlap with CDS regions. In order to verify the SVs, especially insertions and deletions, at a population level, we aligned resequencing data of 12 gray wolves to the dog reference genome, and realized 4863 insertions and 74 389 deletions by breakdancer, and 82 479 insertions and 66 014 deletions by CNVnator. After integrating the two sets by Hugeseq scripts, there were 45 156 insertions and 34 030 deletions left in total. It is worth noting that insertions and deletions in dogs correspond to deletions and insertions in gray wolves. As a result, the overlap ratios of dog deletions to wolf insertions and dog insertions to wolf deletions were 42.3 and 90.5%, respectively. Meanwhile, the overlap ratio of the dog-specific regions to wolf deletions was 85.7%. Together, these results attest to a robust pairwise comparison data set.

**Figure 4.**
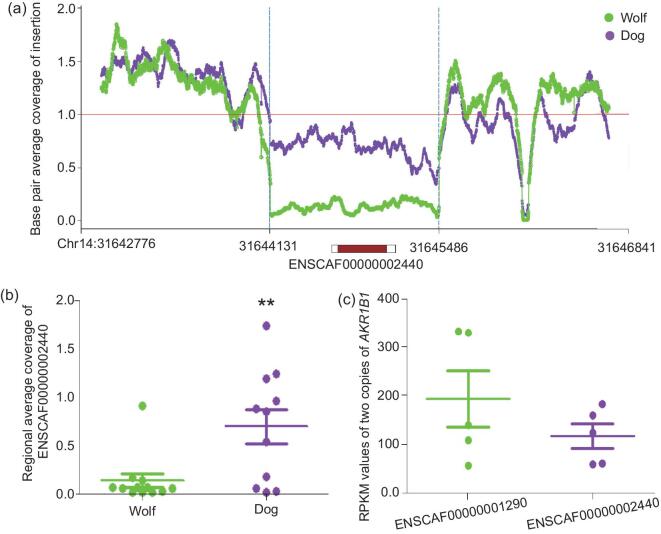
*AKR1B1* (ENSCAFG00000001290) and the dog-specific insertion (chromosome 14:31642776–31646841) in detail. (a) Average coverage flanking the insertion calculated based on each base pair. Two-thirds of this area represent the dog-specific insertion containing ENSCAFG00000002440. Fluorescent green line signifies average read depth of 12 gray wolves and purple line signifies average read depth of 11 dogs. (b) Regional coverage of ENSCAFG00000002440 of dogs and gray wolves. Fluorescent green dots signify regional read depth of ENSCAFG00000002440 in 12 gray wolves and purple dots signify regional read depth of ENSCAFG00000002440 in 11 dogs. The regional read depth of ENSCAFG00000002440 is significantly different between dogs and wolves (Mann–Whitney–Wilcoxon test: *P* = 0.027). (c) Expression of *AKR1B1* (ENSCAFG00000001290) and the new copy (ENSCAFG00000002440) in five Chinese indigenous dogs.

Interestingly, we identified an insertion (chromosome 14:31644131–31645486) that encompasses an intact gene, ENSCAFG00000002440, denoted as a ‘novel gene' in Ensembl annotation (v87). Comparative analysis showed that it is a new copy of *AKR1B1* (Aldo-Keto Reductase Family 1 Member B), a gene encoding an enzyme that catalyzes the rate-limiting reduction of glucose to sorbitol using NAD(P)H as a cofactor [36]. The insertion leads to two copies of *AKR1B1* in the dog genome. To verify this finding, we analyzed the insertion at population level using resequencing data of 11 dogs and 12 gray wolves (see detail in Online Methods). We calculated the average coverage of every base pair in this location for both populations and calculated the average coverage of this regional for each sample. As shown in Fig. 4a, the coverage of the dog-specific insertion is obviously higher in dogs than in gray wolves, while the coverage of the regions flanking the insertion is almost the same across the two populations. As shown in Fig. 4b, the read depth of all but one gray wolf is close to 0, while that of most dogs is nearly 1. Furthermore, Mann–Whitney–Wilcoxon testing shows a significant difference in the regional average coverage between dog and wolf populations (*P* = 0.0268).

To verify whether the new copy of the *AKR1B1* is expressed or not, we sequenced small intestine tissues of five indigenous dogs from China (Supplementary Table S23). Because there is only one different site between the coding sequence of ENSCAFG00000001290 (A at chromosome 14:3003654) and ENSCAFG00000002440 (T at chromosome 14:31644927) in our RNA-sequencing (RNA-seq) libraries, we calculated RPKM (Reads Per Kilobases per Million reads) values in a 200 bp-length (2-fold read length) window, placing the different site at the center, in order to overcome random effects in mapping reads to other regions containing the same sequence. As shown in Fig. 4c, the new copy of *AKR1B1* expresses in the small intestine. On average, the value of RPKM of ENSCAFG00000002440 is 117.4 and the value of ENSCAFG00000001290 is 193.1, suggesting that it could be functional in the small intestine. These results indicated that the copy number gain of the *AKR1B1* gene during dog domestication could be associated with dogs' adaption from a carnivorous diet to a starch diet.

## DISCUSSION

Comparative genomic analysis is a powerful approach for the discovery of SVs as well as copy number variations [37,38]. Supported by deep coverage and accurate read arrangement of the *de novo* assembly, we applied this approach to acquire high-resolution structural SVs, and further enabled the improvement of breakpoint inaccuracy, ambiguous mapping in repetitive regions and length limits inherent in SV calling methods based on resequenced genome alignments [2]. In the present study, we *de novo*-assembled high-quality draft genome sequences from two canine species, the gray wolf (*C*. *lupus*) and dhole (*Cu*. *alpinus*), and describe the evolution of SVs during dog domestication for the first time. We further illustrate that the gray wolf genome holds fundamental evolutionary information that could be missed where mapping is directly done to the dog genome, and that the dhole genome could be used as an outgroup in canine demographic history and phylogenetic research.

The survey of repeated elements showed that the dog genome has more LINEs, particularly two *Canis*-specific TEs, L1_Canis1 and L1_Cf, than the other two canine genomes. As revealed by the highly conserved synteny of these three genomes, the total number of each of these elements in the dog genome is almost two-times greater in dog than both gray wolf and dhole (L1_Canis1: 2.18 of dog/wolf and 2.37 of dog/dhole, L1_Cf: 2.01 of dog/wolf and 2.24 of dog/dhole). The biological functions of L1_Canis1 and L1_Cf are unclear, but L1 insertion into genes could cause genetic defects by altering regulatory and structural properties at the site of insertion [39]. L1 is reported to associate with lamellar ichthyosis and Duchenne-like muscular dystrophy in breed dogs [40,41]. We reason that the genetic variations generated by TEs, for example L1, might be important raw materials for selective breeding programs [42]. Our comparative analysis also showed that a major canine-specific short interspersed element (SINE), SINEC_Cf, has undergone expansion. Previous studies discovered that it features in one-half of all genes in dogs and contributes canine genomic diversity [30,43]. The diversity of the SINEC_Cf repeats is responsible for phenotypes and traits in dog breeds. For instance, a SINEC_Cf insertion in the *IGF1* gene associates with small body size [44], and a SINEC_Cf in the *SILV* gene causes merle patterning [45].

Genes located in dog-specific regions show significant enrichment in categories including olfactory transduction, ribosome, drug metabolism, and starch and sucrose metabolism. Cross analysis of these genes with gene family expansion results still retain the enrichment of olfactory transduction, starch metabolism and immunity categories. More importantly, we identified *AMY2B* as a copy number variation in dog, which has previously been associated with starch digestion in dogs [9]. The identification of *AMY2B* manifests the reliability of our approaches for exploring the genetics of dog domestication. Most interestingly, *AMY2B* gene copies are increased in ancient dog populations of Western and Eastern Europe and Southwest Asia, but not in Australian and Arctic dogs, reflecting the spread of prehistoric agriculture [46] and a local adaptation that allowed dogs to thrive on a starch-rich diet [47].

The evolution of olfactory transduction in dogs is particularly important because it is connected with performance traits that humans selected during the domestication process and the development of specific dog breeds [48]. Differences in selection drive olfactory receptor genes in different directions between dogs and wolves [49]. For instance, the initial level of polymorphism of olfactory receptors was high, leading to amino acid changes and pseudogenization [50], but artificial selection acting on them changed during the domestication of dogs [49]. Thus, the SVs in the categories of olfactory transduction could have evolved in diverse ways during the dog domestication process. Nevertheless, the enrichment of olfactory transduction and immune categories might not only be due to artificial selection during domestication, but may also be caused by genome assembly bias stemming from high sequence similarities of these gene families [51].

As SVs are closely related to phenotypic variation in domestication, we analyzed SVs between the dog and dhole genomes, dog and wolf genomes, and dog-specific regions, which provided comprehensive data regarding SVs related to dog domestication. The results show that deletion and insertion account for the biggest proportion of SVs. Moreover, most SVs are harmful to genes; therefore, CDS regions tend limit SVs to the minimum through negative selection. These two patterns are consistent with what have been observed in other organisms, such as human and fly [52,53]. To eliminate or decrease the assembly noise of wolf and dhole, we focused on SVs in dog-specific regions and phased their potential impacts for dogs' phenotypes. Among genes overlapping with SVs, functional terms including energy metabolism, and neurological and immune processes feature prominently. Also, few other SVs affect the functions of the pigment, olfactory and skeletal systems. Physical and behavioral changes are important consequences of mammalian domestication [54]. Changes in feeding habits, immune systems and reproductive cycles, strong selection on reducing aggressive behavior and neurological traits, and the alteration of body size and coat color are common in domestic animals [7,55,56].

Of particular interest is the copy number gain of a carbohydrate metabolism gene, *AKR1B1*, which we validated at population level. We detected its expression in the small intestine tissues of five indigenous dogs from China, suggesting its functionality. The uncovered 5 bp on the 3′ end of the *AKR1B1* insertion's coordinates and the gene location may be explained by the lack of precision of SV breakpoint detection using next-generation sequencing data. The adaptation of dogs to a starch-rich diet from a mainly carnivorous diet is a significant variation and holds profound implications for its evolution. This alteration of feeding habit allowed dog ancestors to thrive during the agricultural revolution and promulgated the journey of dogs towards successful domestication.

*AKR1B1* is an enzyme that converts glucose to its sugar alcohol form, sorbitol, using NADPH as the reducing agent. *AKR1B1* also displays antioxidant ability by reducing dietary electrophilic carbonyls and protects the small intestine cells from oxidative damage [57]. Moreover, *AKR1B1* and *AKR1B10* are two homologs of the *AKR1B*, and share similar amino acid sequences and 3D structures in humans, which suggests potential functional commonality [58]. Previous studies have demonstrated that *AKR1B10* can increase *de novo* fatty acid synthesis by inhibiting acetyl-CoA carboxylase-α degradation [59,60]. In this study, we postulate that a copy number gain of gene *AKR1B1* may imply that dogs tend to have upregulated *de novo* fatty acid synthesis in the small intestine and liver compared to gray wolves. We also found other genes involved in fatty acid metabolism that overlapped with SVs. For instance, *FASN* (Fatty Acid Synthase) encodes an enzyme that catalyzes the synthesis of palmitate from acetyl-CoA and malonyl-CoA into long-chain saturated fatty acids [61]. This suggests that the high-starch diet during the agricultural revolution not only influenced carbohydrate metabolism [9,62], but also lipid synthesis and carbonyl detoxification [63] in the domestic dog. Besides, another gene, *GALNT7* (Polypeptide N-Acetylgalactosaminyltransferase 7), involved in carbohydrate metabolic processes, was fully covered by an insertion. However, statistical evidence does not sufficiently support the gain of *GALNT7* to be related to dog domestication. Overall, these results illustrate that RNA-based gene duplication generated by retroposition can offer raw genetic material for new genes to facilitate important evolutionary processes like domestication [64].

Artificial selection against aggressive behavior and neurological traits is a crucial step in animal domestication [65,66]. In our study, we found 12 genes related to neurological process-bearing insertions, deletions and repeats. *NOTCH3* is involved in forebrain development [67], *PLD2* in synaptic vesicle recycling [68] and *ARRB2* plays a role in the regulation of synaptic receptors [69]. *CYP46A1* converts brain-secreted cholesterol to 24S-hydroxycholesterol for the liver to catabolize [70], *EFNB3* is important in brain development as well as its maintenance [71], *NES* is required for the survival, renewal and mitogen-stimulated proliferation of neural progenitor cells [72], and *CACNG7* is involved in the transmission of nerve impulses [73]. *MTNR1B* is related to chemical synaptic transmission [74], *PLXNC1* is responsible for the regulation of axon extension involved in axon guidance [75], *DNAH8* is relevant to neuronal migration and development [76], *ACAN* is connected with central nervous system development [77] and *SNAP23* is related to synaptic vesicle priming [78].

Besides protein-coding genes, advances in high-throughput transcriptome sequencing has increasingly illuminated the importance of lincRNAs in evolutionary biology [79]. In our study, we applied new tools and methods [80] to successfully identify 31 lincRNAs in the dog-specific genomic regions in addition to the 215 protein-coding genes. LincRNAs are reported to be involved in hereditary sensory autonomic neuropathy in hunting dogs [81], hence they could be important genetic resources in dog domestication. Furthermore, a recent hypothesis suggested that TEs could be a possible source of functional domains of long non-coding RNAs [82], suggesting the plausibility of integrating canine-specific TEs and lincRNAs to advance our understanding of the phenotypes and diseases of dogs.

## CONCLUSION

The present study reveals that large-scale genetic variants are of great importance and are worth paying more attention to in domestication studies. Our findings broaden our understanding of dog evolution, and provide valuable insights into the vital role and evolutionary dynamics of SVs in the process of dog domestication. Recent artificial selection has produced complex phenotypes and behaviors in dogs resulting in numerous breeds. Increased attention on the evolution of SVs in recent breeding activities, genome-wide association studies and SV-associated QTL mapping among other genomic investigations remains a great necessity. This could shed new light on the genomic basis of complex traits and diseases, including cancer, in dogs.

## METHODS

### Ethical approval

The gray wolf (*C*. *lupus*) sample was collected from Shandong province, China, and the specimen of dhole (*Cu*. *alpinus*) was sampled from Beijing Zoo, China. All experimental protocols pertaining to animals have been reviewed and approved by the internal review board of the Kunming Institute of Zoology, Chinese Academy of Sciences.

### Genome sequencing and assembly

Total genomic DNA was extracted from the blood or tissue samples of the animals using the phenol/chloroform method. The DNA was fragmented and purified by electrophoresis for whole-genome sequencing. Libraries consisting of short paired-end inserts (170–800 bp) and long mate-paired inserts (800 bp to 20 kb) were constructed for genome sequencing according to the Illumina protocol. All libraries were sequenced on the HiSeq 2000 platform (Supplementary Tables S1 and S2 for the gray wolf and dhole, respectively).

Both genomes were assembled to contigs by the paired-end reads using anytag [83] and Newbler [84]. The paired-end short reads were converted into near error-free pseudo-Sanger sequences by anytag with the parameter ‘Anytag-2.5.2-g 3000000000 -X 50'. Newbler was used to assemble the pseudo-Sanger sequences into contig sets with the parameter ‘runAssembly -large -het -m -noace –nobig'. The mate-pair reads were used to join the contigs into scaffolds by SSPACE [85], and the remaining gaps within these scaffolds were iteratively filled with paired-end reads using GapCloser with the default parameters [86]. Lastly, the short reads were mapped back to the gap-closing scaffolds using the Burrows–Wheeler Aligner (BWA) alignment program [87,88], and VCF files were processed by SAMTools [89].

### Repeat identification, gene prediction and annotation

Repeated elements were annotated by the homology-based approach using RepeatMasker in conjunction with the known repeat library [90]. Tandem Repeats Finder was used to detect tandem repeats in the genomic sequence data [91]. Transcriptome data from multiple tissues (the liver, tongue, olfactory bulb and caudate nucleus) of the gray wolf were aligned to the genome using Tophat [92] and assembled using cufflinks [93]. *De novo* predictions and homolog-based predictions were integrated to annotate the protein-coding genes in both genomes. *De novo* prediction was performed based on the repeat-masked genome using four approaches: Augustus [94], GENSCAN [95], GlimmerHMM [96] and SNAP [97]. Homolog-based prediction was performed through TblastN [98] and GeneWise [99]. The Expressed Sequence Tag (EST) of Carnivora was aligned by PASA [100] to link the spliced alignments and predict possible gene models. The final gene sets of the two species were assessed by BUSCO with mammalian gene sets [31]. All gene evidence predicted sets were combined by EvidenceModeler [101]. Gene functions of protein-coding genes were annotated based on the best hit to two integrated protein sequence databases by BLASTp [98]. Gene motifs and domains were identified by the InterProScan against protein databases [102].

### Expansion and contraction analysis of gene families

Gene families were identified using TreeFam [103]. All the protein sequences of nine species (human, mouse, dog, cow, cat, horse, chicken, wolf and dhole) were searched in TreeFam (version 9) HMM file using hmmsearch 3.1, with the best search result and E value cutoff ≤ 1E-10 after adjusting the gene number in each TreeFamily. All proteins in one family were aligned by the muscle program with default parameters, and the tree built using treebest based on CDSs transformed from the protein sequences. The numbers of proteins of each species in each gene family were collected, and the expansion and contraction of the orthologous gene families determined by comparing the cluster size differences between dhole, wolf, dog and five other mammals using the CAFE program (version 2.2) [104]. A random birth and death process was used to study changes of gene families along each lineage of the phylogenetic tree that we specified. The birth–death parameter λ was estimated using an optimization algorithm, where CAFE starts with an intermediate value and then searches iteratively for the best value for λ that maximizes the log likelihood of the data for all families. A conditional *P*-value and false discovery rate correction were calculated for each gene family, and families with conditional *P*-values under the threshold (0.05) were considered to have accelerated rates of gain or loss.

### Whole-genome alignment of dog, gray wolf and dhole genomes

LASTZ [105] and multiz [106] were carried out on the three Caninae genomes. Each genome sequence was aligned to the other two genomes by LASTZ, with the parameter ‘lastz target [query] M = 254 K = 4500 L = 3000 Y = 15000 T = 2 –format = lav'. The alignments were converted to axt format using the program lavToAxt. axtChain formed maximally scoring chains out of the gapless subsections of the input alignments (with the parameter ‘-linearGap = medium'). chainPreNet and chainNet were used to form a hierarchy of chains that we called a net. The output net files were further annotated by the program netSyntenic with the default settings. Subsequently, the netToAxt, axtSort and axtToMaf programs were used to change net to MAF format, which is a multiple alignment format developed at University of California Santa Cruz, with exactly two sequences per block in which the first row comes from the target sequence and the second from the query [107]. Finally, we performed multiple alignments among the three genomes based on the result of pairwise alignment (MAF files) and produced an evolutionary tree with the program multiz-tba.012109 with command ‘tba “((dhole wolf) dog)” maf-source tba.maf'. The set of SVs was extracted using in-house Perl scripts, based on the condition of the three alignment pairs.

Gene ontology enrichment and functional annotation of genes linked with dog-specific SVs was implemented by DAVID [108]. The enrichment thresholds score was set to 0.05 and the Benjamini– Hochberg method was chosen to correct *P*-values. Gene clusters with *P*-values < 0.05 were considered significant.

### Verification of dog and gray wolf populations

A total of 12 gray wolf genomes were downloaded for population-level analysis of SVs [109]. Raw sequence reads of each individual were mapped to the dog reference genome (Canfam3) [16] using BWA [87] with default parameters. SAMtools (v.0.1.18) was used to sort and remove PCR duplicates [89]. To minimize false positive SNP calls around indels, local realignment around indels was performed using the Genome Analysis Tool Kit [110]. Breakdancer [111] and CNVnator [112] were used to detect SVs. For Breakdancer, we set the minimum number of read pairs required for an SV to four and minimum mapping quality to 35. For CNVnator, we used a bin size of 300 bp. All SVs shorter than 50 were filtered and the results from these two SV calling sets were integrated using Shell scripts provided by Hugeseq [113]. Cross-checking with pairwise SVs was accomplished by in-house Perl scripts.

We checked the average base pair coverage of the insertion (chromosome 14:31644131–31645486) at population level through published data of 11 dogs and 12 gray wolves [109]. We used the average coverage of two marginal regions (of the same length as the insertion) of this insertion for comparison. The raw read depth of the whole region (chromosome 14:31642776–31646841) was determined by SAMtools [89] from each sample. The read depth of base pairs that have not been sequenced was denoted as 0.

We first normalized the read depth of each base by dividing its raw data with the corresponding sample's sequencing depth. The normalized read depth of the same species (gray wolf and dog) was then added base by base and normalized again by the total number of the samples belonging to the species. To calculate the regional average coverage of *AKR1B1*, we did the same procedure as above to get the normalization base pair read depth. We then added the normalized read depth in the *AKR1B1* region and divided it by the length of this gene. Mann–Whitney–Wilcoxon testing of the regional average coverage of *AKR1B1* was conducted in R (version 3.31).

### Transcriptomic analysis

Specimens of small intestine of five indigenous dogs were collected from Kunming, China for transcriptome sequencing. Total RNA was extracted from each tissue using the TRIzol kit (Life Technologies). Libraries were constructed and sequenced according to the Illumina protocol. After adapter trimming of the RNA-seq library by cutadapt [114] and filtering of residual reads by TrimmomaticPE with parameters LEADING:3 TRAILING:3 SLIDINGWINDOW:4:15 MINLEN:36 [115], we aligned the high-quality RNA-seq reads from dog small intestine to the Canfam3 reference genome with Ensemble release 75 annotation using Tophat with default parameters [116]. Expression levels of each gene in each RNA-seq library were measured using Cufflinks with default parameters [93].

## Supplementary Material

Supplementary Files

## ACCESSION CODES

The raw sequence data from this study have been submitted to the Genome Sequence Archive (http://gsa.big.ac.cn/) under accession CRA000199 for raw data of genomes, CRA000955 for raw data of the dog transcriptome and CRA000973 for raw data of the wolf transcriptome. The genome assemblies have been deposited in the Genome Warehouse in the BIG Data Center (http://bigd.big.ac.cn/gwh) under accession number PRJCA000364 (SAMC010682 for *C. lupus* and SAMC010683 for *Cu. alpinus*). Multiple alignments and SVs have been submitted to the DogSD database (DogSD: http://dogsd.big.ac.cn).
