# Supplementary material for: Structural variation during dog domestication: insights from gray wolf and dhole genomes
Source: Natl Sci Rev. 2018 Jul 19;6(1):110–22. doi: 10.1093/nsr/nwy076 (PMC8291444; doi:10.1093/nsr/nwy076)
Supplement: Supplementary Files [file nwy076_supplemental_file.doc]

**Supplementary Information for**

**Structural variation during dog domestication: insights from grey wolf and dhole genomes.**

Guo-Dong Wang^1,2#^, Xiu-Juan Shao^3#^, Bing Bai^4,5#^, Junlong Wang^7,8^, Xiaobo Wang^3^, Xue Cao^8^, Yan-Hu Liu^9^, Xuan Wang^1,10^, Ting-Ting Yin^1,10^, Shao-Jie Zhang^9^, Yan Lu^11^, Zechong Wang^11^, Lu Wang^9^, Wenming Zhao^12^, Bing Zhang^12^, Jue Ruan^3*^, and Ya-Ping Zhang^1,2*^

^1^State Key Laboratory of Genetic Resources and Evolution, Kunming Institute of Zoology, Chinese Academy of Sciences, Kunming 650223, China;

^2^Center for Excellence in Animal Evolution and Genetics, Chinese Academy of Sciences, Kunming 650223, China;

^3^Agricultural Genomics Institute, Chinese Academy of Agricultural Sciences, Shenzhen 518120, China;

^4^The genetics and diagnosis center of the first hospital of Yunnan province, Kunming, 650032, China;

^5^Department of Pediatrics, the First People's Hospital of Yunnan Province, Kunming, Yunnan 650032, China;

^6^College of Pharmacology, Soochow University, Suzhou 215123, China;

^7^Key Laboratory of Animal Models and Human Disease Mechanisms of the Chinese Academy of Sciences & Yunnan province, Kunming Institute of Zoology, Chinese Academy of Sciences, Kunming, 650223, China;

^8^Department of Laboratory Animal Science, Kunming Medical University, Kunming 650500, China;

^9^Laboratory for Conservation and Utilization of Bio-Resources & Key Laboratory for Microbial Resources of the Ministry of Education, Yunnan University, Kunming 650091, China;

^10^Kunming College of Life Science, University of Chinese Academy of Sciences, Kunming 650204, China;

^11^Beijing Zoo, Beijing 100044, China;

^12^Core Genomic Facility, Beijing Institute of Genomics, Chinese Academy of Sciences, Beijing 100101, China;

#These authors contributed equally to this work.

*Correspondence:

Ya-Ping Zhang (zhangyp@mail.kiz.ac.cn) OR Jue Ruan ([ruanjue@gmail.com](mailto:ruanjue@gmail.com)).

The authors declare no conflict of interest.

This file includes

Supplementary figure 1-2

Supplementary table S1-S23


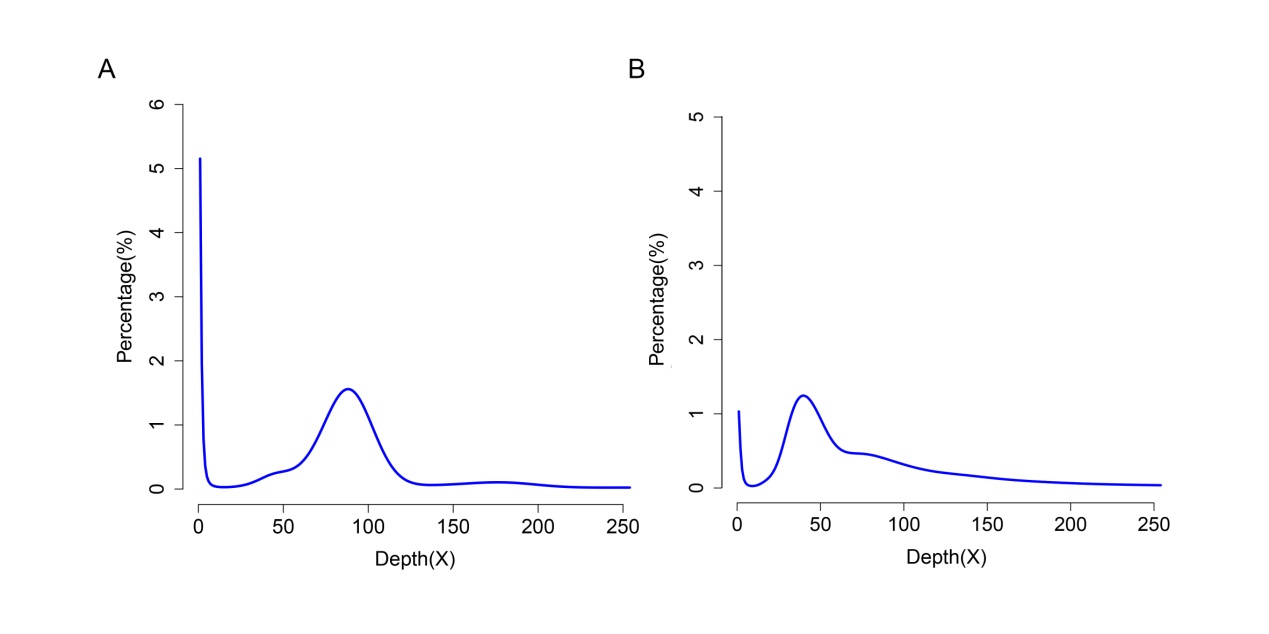


Supplementary figure 1. The K-mer frequency distribution of two canine genomes. A) 19-mer frequency distribution of the grey wolf genome. B) 17-mer frequency distribution of the dhole genome.


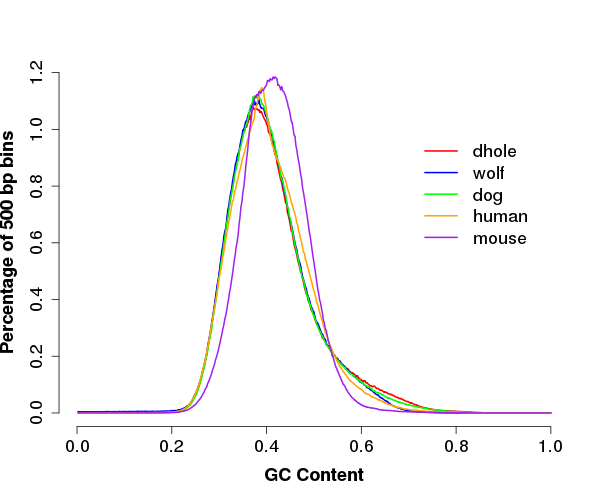


Supplementary figure 2. Comparison of GC content of five mammalian species. The x-axis indicates GC content and the y-axis indicates the percentage of the bin number by sliding 500bp non-overlapping windows against the whole genome.

Supplementary table 1. Summary of sequenced data of the grey wolf genome.

| **Libraries** | **Insert size**  **(bp)** | **Read length**  **(bp)** | **Raw reads** | | **Qualified reads** | |
| --- | --- | --- | --- | --- | --- | --- |
|  |  |  | **Total data(Gb)** | **Sequence coverage(X)** | **Total data(Gb)** | **Sequence coverage(X)** |
| **Illumina reads** | 170bp | 104 | 38.6 | 16.0 | 28.6 | 11.9 |
|  | 300bp | 104 | 49.6 | 20.6 | 45.1 | 18.7 |
|  | 400bp | 104 | 48.8 | 20.3 | 44.2 | 18.4 |
|  | 500bp | 92 | 43 | 17.9 | 39 | 16.2 |
|  | 600bp | 110 | 49.4 | 20.5 | 43.4 | 18.0 |
|  | 800bp_PE | 104 | 37.4 | 15.5 | 30.7 | 12.7 |
|  | 800bp_MP | 85 | 26.7 | 11.1 | 26.5 | 11.0 |
|  | 2Kb | 49 | 22.7 | 9.4 | 22.1 | 9.2 |
|  | 5Kb | 49 | 14.5 | 6.0 | 13.9 | 5.8 |
|  | 10kb | 49 | 11 | 4.6 | 10.3 | 4.3 |
|  | 20kb | 49 | 7.9 | 3.3 | 7.3 | 3.0 |
| **Total** | - | - | 349.7 | 145.2 | 311.1 | 129.2 |

Supplementary table 2. Summary of sequenced data of the dhole genome.

| **Libraries** | **Insert size**  **(bp)** | **Read length**  **(bp)** | **Raw reads** | | **Qualified reads** | |
| --- | --- | --- | --- | --- | --- | --- |
|  |  |  | **Total data(Gb)** | **Sequence coverage(X)** | **Total data(Gb)** | **Sequence coverage(X)** |
| **Illumina reads** | 200bp | 150 | 36.5 | 13.9 | 35.6 | 13.5 |
|  | 500bp | 150 | 42.7 | 16.2 | 40.3 | 15.3 |
|  | 650bp | 92 | 29.2 | 11.1 | 29.2 | 11.1 |
|  | 800bp | 86 | 20.6 | 7.8 | 20.6 | 7.8 |
|  | 3Kb | 95 | 27 | 10.3 | 26.9 | 10.2 |
|  | 8kb | 96 | 20.3 | 7.7 | 20.3 | 7.7 |
|  | 15kb | 97 | 37.1 | 14.1 | 37 | 14.1 |
| **Total** | - | - | 213.4 | 81.1 | 209.9 | 79.7 |

Supplementary table 3. Repeat annotation in the grey wolf genome.

| **File name** | wolf.genome.fa | | | |
| --- | --- | --- | --- | --- |
| **Sequences** | 44203 | | | |
| **Total length** | 2313148660 bp(2259454654 excl N/X-runs) | | | |
| **GC level** | 40.70% | | | |
| **Bases masked** | 908176566 bp(39.26%) | | | |
|  |  | number of elments* | length occupied (bp) | percentage of sequence (%) |
| **SINEs** | | 1470906 | 240411925 | 10.39 |
|  | **Alu/B1** | 0 | 0 | 0 |
|  | **MIRs** | 428181 | 63303252 | 2.74 |
| **LINEs** | | 872121 | 437460733 | 18.91 |
|  | **LINE1** | 532302 | 348633039 | 15.07 |
|  | **LINE2** | 289070 | 77403890 | 3.35 |
|  | **L3/CR1** | 37523 | 8218057 | 0.36 |
|  | **RTE** | 12054 | 3015643 | 0.13 |
| **LTR elements** | | 299903 | 115575671 | 5 |
|  | **ERVL** | 88172 | 39234773 | 1.7 |
|  | **ERVL-MaLRs** | 142926 | 49322218 | 2.13 |
|  | **ERV_classI** | 46955 | 21558506 | 0.93 |
|  | **ERV_classII** | 0 | 0 | 0 |
| **DNA elements** | | 318218 | 67024759 | 2.9 |
|  | **hAT-Charlie** | 184506 | 35787295 | 1.55 |
|  | **TcMar-Tigger** | 47852 | 14280629 | 0.62 |
| **Unclassified** | | 6258 | 1106928 | 0.05 |
| **Total interspersed repeats** | |  | 861580016 | 37.25 |
| **Small RNA** | | 1080599 | 179717502 | 7.77 |
| **Satellites** | | 871 | 844231 | 0.04 |
| **Simple repeats** | | 833484 | 36826283 | 1.59 |
| **Low complexity** | | 113493 | 5738809 | 0.25 |

Supplementary table 4. Repeat annotation in the dhole genome.

| **File name** | dhole.genome.fa | | | |
| --- | --- | --- | --- | --- |
| **Sequences** | 29680 | | | |
| **Total length** | 2329418464 bp(2288176676 excl N/X-runs) | | | |
| **GC level** | 41.26% | | | |
| **Bases masked** | 897083571 bp(38.51%) | | | |
|  |  | number of elments* | length occupied (bp) | percentage of sequence (%) |
| **SINEs** | | 1446973 | 235838516 | 10.12 |
|  | **Alu/B1** | 0 | 0 | 0 |
|  | **MIRs** | 428868 | 63375151 | 2.72 |
| **LINEs** | | 866044 | 431335288 | 18.52 |
|  | **LINE1** | 526205 | 342639939 | 14.71 |
|  | **LINE2** | 289026 | 77287911 | 3.32 |
|  | **L3/CR1** | 37593 | 8211240 | 0.35 |
|  | **RTE** | 12052 | 3006930 | 0.13 |
| **LTR elements** | | 298212 | 114678739 | 4.92 |
|  | **ERVL** | 87566 | 39001817 | 1.67 |
|  | **ERVL-MaLRs** | 142393 | 49138520 | 2.11 |
|  | **ERV_classI** | 46543 | 21114026 | 0.91 |
|  | **ERV_classII** | 0 | 0 | 0 |
| **DNA elements** | | 317418 | 66779281 | 2.87 |
|  | **hAT-Charlie** | 184014 | 35669315 | 1.53 |
|  | **TcMar-Tigger** | 47699 | 14223951 | 0.61 |
| **Unclassified** | | 6252 | 1105459 | 0.05 |
| **Total interspersed repeats** | |  | 849737283 | 36.48 |
| **Small RNA** | | 1056058 | 179717502 | 7.77 |
| **Satellites** | | 540 | 460206 | 0.02 |
| **Simple repeats** | | 873706 | 37581714 | 1.61 |
| **Low complexity** | | 121639 | 6118461 | 0.26 |

Supplementary table 5. Repeat annotation in the dog reference genome.

| **File name** | CanFam3.1.dna.fa | | | |
| --- | --- | --- | --- | --- |
| **Sequences** | 40 | | | |
| **Total length** | 2327650711(2317672501 bpexcl N/X-runs) | | | |
| **GC level** | 41.06% | | | |
| **Bases masked** | 947195749 bp( 40.69 %) | | | |
|  |  | number of elments* | length occupied (bp) | percentage of sequence (%) |
| **SINEs** | | 1485109 | 243825728 | 10.48 |
|  | **Alu/B1** | 0 | 0 | 0 |
|  | **MIRs** | 428958 | 63419463 | 2.72 |
| **LINEs** |  | 862434 | 471625447 | 20.26 |
|  | **LINE1** | 522548 | 382928013 | 16.45 |
|  | **LINE2** | 288967 | 77250913 | 3.32 |
|  | **L3/CR1** | 37634 | 8249008 | 0.35 |
|  | **RTE** | 12116 | 3010287 | 0.13 |
| **LTR elements** | | 298195 | 115463691 | 4.96 |
|  | **ERVL** | 87874 | 39219618 | 1.68 |
|  | **ERVL-MaLRs** | 142464 | 49235846 | 2.12 |
|  | **ERV_classI** | 46096 | 21564178 | 0.93 |
|  | **ERV_classII** | 0 | 0 | 0 |
| **DNA elements** | | 317789 | 67023654 | 2.88 |
|  | **hAT-Charlie** | 184167 | 35787189 | 1.54 |
|  | **TcMar-Tigger** | 47707 | 14284694 | 0.61 |
| **Unclassified** | | 6265 | 1108676 | 0.05 |
| **Total interspersed repeats** | |  | 899047196 | 38.62 |
| **Small RNA** | | 1094165 | 183028607 | 7.86 |
| **Satellites** | | 481 | 622134 | 0.03 |
| **Simple repeats** | | 892236 | 38314936 | 1.65 |
| **Low complexity** | | 120614 | 6012773 | 0.26 |

Supplementary table 6. TE annotations in the grey wolf genome based on de novo TE database.

|  | Repbase TEs | | RepeatModeler TEs | | Combined TEs | |
| --- | --- | --- | --- | --- | --- | --- |
|  | Length(bp) | % | Length(bp) | % | Length(bp) | % |
| DNA | 67,024,759 | 2.90 | 37,800,060 | 1.63 | 70,393,136 | 3.05 |
| LINE | 437,460,733 | 18.91 | 481,747,672 | 20.83 | 590,432,195 | 25.56 |
| LTR | 115,575,671 | 5.00 | 74,957,087 | 3.24 | 118,487,722 | 5.13 |
| SINE | 240,411,925 | 10.39 | 63,116,733 | 2.73 | 121,547,729 | 5.26 |
| Other | 223,126,825 | 9.64 | 78,657,017 | 3.40 | 42,001,817 | 1.82 |
| Unknown | 1,106,928 | 0.05 | 2,623,052 | 0.11 | 3,055,514 | 0.13 |
| Total | 861,580,016 | 37.25 | 660,244,604 | 28.54 | 903,916,296 | 39.13 |

Supplementary table 7. TE annotations in the dhole genome based on de novo TE database.

|  | Repbase TEs | | RepeatModeler TEs | | Combined TEs | |
| --- | --- | --- | --- | --- | --- | --- |
|  | Length(bp) | % | Length(bp) | % | Length(bp) | % |
| DNA | 66,779,281 | 2.87 | 39,184,893 | 1.68 | 71,133,293 | 3.06 |
| LINE | 431,335,288 | 18.52 | 450,323,076 | 19.33 | 559,224,422 | 24.08 |
| LTR | 114,678,739 | 4.92 | 78,236,784 | 3.36 | 122,164,974 | 5.26 |
| SINE | 235,838,516 | 10.12 | 81,174,912 | 3.48 | 139,035,178 | 5.99 |
| Other | 219,231,829 | 9.41 | 97,136,347 | 4.17 | 42,298,185 | 1.82 |
| Unknown | 1,105,459 | 0.05 | 2,555,070 | 0.11 | 2,892,212 | 0.12 |
| Total | 849,737,283 | 36.48 | 651,474,735 | 27.97 | 894,450,079 | 38.51 |

Supplementary table 8. TE annotations in the dog genome based on de novo TE database.

|  | Repbase TEs | | RepeatModeler TEs | | Combined TEs | |
| --- | --- | --- | --- | --- | --- | --- |
|  | Length(bp) | % | Length(bp) | % | Length(bp) | % |
| DNA | 67,333,760 | 2.79 | 38,272,512 | 1.59 | 70,788,273 | 2.94 |
| LINE | 486,212,802 | 20.17 | 516,754,403 | 21.43 | 621,441,557 | 25.78 |
| LTR | 117,570,418 | 4.88 | 85,842,032 | 3.56 | 129,410,449 | 5.37 |
| SINE | 246,828,158 | 10.24 | 90,708,819 | 3.76 | 145,541,320 | 6.04 |
| Other | 270,147,284 | 11.20 | 110,897,300 | 4.60 | 83,426,683 | 3.46 |
| Unknown | 1,110,955 | 0.05 | 6,259,863 | 0.26 | 4,776,225 | 0.20 |
| Total | 919,056,093 | 38.12 | 737,837,629 | 30.60 | 971,957,824 | 40.31 |

Supplementary table 9. LINE contents in three Canid genomes.

| sub_class | class | dog | wolf | dhole |
| --- | --- | --- | --- | --- |
| HAL1 | LINE/L1 | 6082582 | 5999240 | 5978679 |
| HAL1b | LINE/L1 | 958521 | 950772 | 941827 |
| HAL1M8 | LINE/L1 | 669398 | 667323 | 677805 |
| HAL1ME | LINE/L1 | 1335986 | 1308644 | 1297649 |
| L1_Canid_ | LINE/L1 | 14420953 | 13224994 | 12864093 |
| L1_Canid2 | LINE/L1 | 3902975 | 3709460 | 3645582 |
| L1_Canis1 | LINE/L1 | 65757351 | 30116012 | 27781790 |
| L1_Canis2 | LINE/L1 | 5041715 | 7270039 | 6577792 |
| L1_Carn1 | LINE/L1 | 4494219 | 3180170 | 3167494 |
| L1_Carn2 | LINE/L1 | 17411981 | 15506963 | 15437387 |
| L1_Carn3 | LINE/L1 | 21629837 | 21468071 | 21139568 |
| L1_Carn5 | LINE/L1 | 13435909 | 13380446 | 13375475 |
| L1_Carn7 | LINE/L1 | 20553478 | 20201136 | 20071801 |
| L1_Carni | LINE/L1 | 698393 | 704975 | 679543 |
| L1_Cf | LINE/L1 | 13740230 | 6842615 | 6121615 |
| L1M | LINE/L1 | 66204 | 67478 | 63976 |
| L1M2 | LINE/L1 | 535196 | 570590 | 563221 |
| L1M3 | LINE/L1 | 3133527 | 2836496 | 2844475 |
| L1M3a | LINE/L1 | 165724 | 172563 | 173947 |
| L1M3b | LINE/L1 | 255598 | 246348 | 261195 |
| L1M3c | LINE/L1 | 667251 | 648351 | 676038 |
| L1M3d | LINE/L1 | 174615 | 178061 | 172684 |
| L1M3de | LINE/L1 | 135135 | 139688 | 122764 |
| L1M3e | LINE/L1 | 112579 | 104035 | 120920 |
| L1M4 | LINE/L1 | 6456756 | 6432380 | 6482902 |
| L1M4a1 | LINE/L1 | 914585 | 976511 | 967955 |
| L1M4a2 | LINE/L1 | 712684 | 700095 | 693874 |
| L1M4b | LINE/L1 | 1714506 | 1712719 | 1691146 |
| L1M4c | LINE/L1 | 1171715 | 1145516 | 1148061 |
| L1M5 | LINE/L1 | 10953566 | 10974584 | 10897777 |
| L1M6 | LINE/L1 | 996895 | 989133 | 997828 |
| L1M6B | LINE/L1 | 72943 | 74061 | 69757 |
| L1M7 | LINE/L1 | 796822 | 782062 | 780865 |
| L1M8 | LINE/L1 | 527762 | 525298 | 529281 |
| L1MA10 | LINE/L1 | 1221009 | 1184042 | 1221998 |
| L1MA9 | LINE/L1 | 13654538 | 13725952 | 13534065 |
| L1MB1 | LINE/L1 | 2271155 | 2283012 | 2229698 |
| L1MB2 | LINE/L1 | 2838508 | 2774468 | 2777249 |
| L1MB3 | LINE/L1 | 5774943 | 5787488 | 5752990 |
| L1MB4 | LINE/L1 | 2890150 | 2783490 | 2796859 |
| L1MB5 | LINE/L1 | 3247792 | 3242621 | 3171774 |
| L1MB7 | LINE/L1 | 8599551 | 8535961 | 8404952 |
| L1MB8 | LINE/L1 | 5874689 | 5785486 | 5783422 |
| L1MC | LINE/L1 | 2360919 | 2367140 | 2369323 |
| L1MC1 | LINE/L1 | 8573928 | 8460149 | 8440016 |
| L1MC2 | LINE/L1 | 2498381 | 2491445 | 2479538 |
| L1MC3 | LINE/L1 | 6275622 | 5504234 | 5419995 |
| L1MC4 | LINE/L1 | 6693932 | 6616537 | 6565166 |
| L1MC4a | LINE/L1 | 2904281 | 2913345 | 2907013 |
| L1MC5 | LINE/L1 | 3758087 | 3770188 | 3746383 |
| L1MC5a | LINE/L1 | 4076935 | 4106699 | 4079735 |
| L1MCa | LINE/L1 | 3725775 | 3727752 | 3705999 |
| L1MCb | LINE/L1 | 1023073 | 1064481 | 1018200 |
| L1MCc | LINE/L1 | 1020707 | 1032096 | 1001497 |
| L1MD | LINE/L1 | 2651845 | 2629977 | 2636623 |
| L1MD1 | LINE/L1 | 3014305 | 3038646 | 3038201 |
| L1MD2 | LINE/L1 | 4133727 | 4076127 | 4075406 |
| L1MD3 | LINE/L1 | 1272813 | 1286515 | 1240205 |
| L1MDa | LINE/L1 | 3050490 | 3027005 | 2973665 |
| L1MDb | LINE/L1 | 373282 | 356853 | 363510 |
| L1ME1 | LINE/L1 | 12098206 | 12012504 | 11886672 |
| L1ME2 | LINE/L1 | 4554451 | 4519554 | 4524772 |
| L1ME2z | LINE/L1 | 1946499 | 1938054 | 1889333 |
| L1ME3 | LINE/L1 | 2590479 | 2565354 | 2542787 |
| L1ME3A | LINE/L1 | 4875027 | 4816899 | 4810169 |
| L1ME3B | LINE/L1 | 2286076 | 2256111 | 2222873 |
| L1ME3C | LINE/L1 | 783377 | 792824 | 766344 |
| L1ME3Cz | LINE/L1 | 3375625 | 3346679 | 3292733 |
| L1ME3D | LINE/L1 | 1098102 | 1098452 | 1072299 |
| L1ME3E | LINE/L1 | 1151725 | 1149184 | 1192509 |
| L1ME3F | LINE/L1 | 1194848 | 1189866 | 1206488 |
| L1ME3G | LINE/L1 | 3368295 | 3386796 | 3308946 |
| L1ME4a | LINE/L1 | 4725759 | 4708879 | 4683549 |
| L1ME4b | LINE/L1 | 4768017 | 4750758 | 4761807 |
| L1ME4c | LINE/L1 | 1512894 | 1513634 | 1503057 |
| L1ME5 | LINE/L1 | 792745 | 797512 | 794592 |
| L1MEa | LINE/L1 | 111994 | 112282 | 101824 |
| L1MEb | LINE/L1 | 469873 | 471160 | 471529 |
| L1MEc | LINE/L1 | 8225447 | 6871344 | 6941116 |
| L1MEd | LINE/L1 | 4744948 | 4739640 | 4741995 |
| L1MEf | LINE/L1 | 4515657 | 4588015 | 4532702 |
| L1MEg | LINE/L1 | 4300322 | 4219636 | 4173126 |
| L1MEg1 | LINE/L1 | 224834 | 228328 | 225240 |
| L1MEg2 | LINE/L1 | 198399 | 189172 | 194770 |
| L1MEh | LINE/L1 | 685746 | 680343 | 665796 |
| L1MEi | LINE/L1 | 1235441 | 1217999 | 1230267 |
| L1MEj | LINE/L1 | 554741 | 567812 | 559891 |
| L1P5 | LINE/L1 | 5335 | 6000 | 6143 |
| L1PB4 | LINE/L1 | 26974 | 42275 | 40726 |
| X9_LINE | LINE/L1 | 32004 | 31029 | 32030 |

Supplementary table 10. Summary of the predicted protein-coding genes in the grey wolf genome.

|  | **Gene set** | **Gene number** | **Total CDS length(Mb)** | **Average CDS length(bp)** | **Exon number pergene** | **Average exon length (bp)** | **Average intron length (bp)** |
| --- | --- | --- | --- | --- | --- | --- | --- |
| **homolog** | Human | 19327 | 30.0 | 1554 | 9.12 | 170 | 2831 |
|  | Mouse | 15135 | 25.6 | 1693 | 10.07 | 168 | 2874 |
|  | Dog | 24806 | 32.1 | 1293 | 7.49 | 173 | 2834 |
| **De novo** | Augustus | 19962 | 29.62 | 1483 | 9.00 | 164.82 | 6628 |
|  | Genescan | 41250 | 51.56 | 1249 | 7.62 | 163.94 | 5629 |
|  | SNAP | 108553 | 83.40 | 768 | 5.32 | 144.52 | 2773 |
|  | GlimmerHMM | 145036 | 150.24 | 1035 | 2.98 | 348.03 | 3149 |
| **RNA-seq** | Tophat+Cufflink | 83434 | 193.39 | 2,317 | 4.94 | 469 | 4,689 |
| **Final gene set** |  | 20797 | 32.3 | 1556 | 9.23 | 169 | 4022 |

Supplementary table 11. Summary of the predicted protein-coding genes in the dhole genome.

|  | **Gene set** | **Gene number** | **Total CDS length(Mb)** | **Average CDS length(bp)** | **Exon number per gene** | **Average exon length(bp)** | **Average intron length (bp)** |
| --- | --- | --- | --- | --- | --- | --- | --- |
| **homolog** | Human | 20067 | 32.5 | 1620 | 9.13 | 177 | 2863 |
|  | Mouse | 16020 | 28.0 | 1745 | 9.95 | 175 | 2902 |
|  | Dog | 26033 | 34.5 | 1324 | 7.39 | 179 | 2870 |
| **De novo** | Augustus | 39230 | 50.49 | 1287 | 5.33 | 241.58 | 6050 |
|  | Genescan | 51829 | 73.59 | 1419 | 6.80 | 208.80 | 4998 |
|  | SNAP | 78621 | 58.06 | 738 | 4.44 | 166.37 | 11408 |
|  | GlimmerHMM | 170024 | 184.48 | 1085 | 2.91 | 372.45 | 2679 |
| **Final gene set** |  | 20045 | 33.5 | 1674 | 9.56 | 175 | 3772 |

Supplementary table 12. Proteomic prediction and functional annotation of the grey wolf and dhole genome, respectively.

|  | **Dhole** | | **Grey wolf** | |
| --- | --- | --- | --- | --- |
|  | **Number** | **Percent(%)** | **Number** | **Percent(%)** |
| **Total** | 20045 | -- | 20797 | -- |
| **Annotated** | 19256 | 96.06 | 18887 | 90.82 |
| **SwissProt** | 18995 | 94.76 | 18472 | 88.82 |
| **TrEMBL** | 19214 | 95.85 | 18762 | 90.21 |
| **InterPro** | 18677 | 93.18 | 18167 | 87.35 |
| **KEGG** | 19159 | 95.58 | 18805 | 90.42 |
| **GO** | 13459 | 67.14 | 12753 | 61.32 |
| **Unannotated** | 789 | 3.94 | 1910 | 9.18 |

Supplementary table 13. BUSCO results based on the presences of 4,104 BUSCO genes (dataset: mammalian odb9).

|  | **Complete** | **Fragmented** | **Missing** |
| --- | --- | --- | --- |
| dhole genes | 3,933(95.8%) | 106(2.6%) | 65(1.6%) |
| wolf genes | 3,760(91.6%) | 192(4.7%) | 152(3.7%) |

Supplementary table 14. Statistics of CDS with Canidae EST mapping (Identity>=95%).

| **Grey wolf** | | **Dhole** | | **Dog** | |
| --- | --- | --- | --- | --- | --- |
| gene number | gene ratio | gene number | gene ratio | gene number | gene ratio |
| 13713 | 66% | 13676 | 68% | 14236 | 71.70% |

Supplementary table 15. Statistics of peptide sequences with 248 human CEGs alignment.

| **Grey wolf** | | **Dhole** | | **Dog** | |
| --- | --- | --- | --- | --- | --- |
| #aligned CEGs | ratio | #aligned CEGs | ratio | #aligned CEGs | ratio |
| 153 | 61.70% | 156 | 62.90% | 170 | 68.50% |

Supplementary table 16. Genome wide synteny of the three canine genomes.

| dog chromomes | chromosme size | dhole | | | grey wolf | | |
| --- | --- | --- | --- | --- | --- | --- | --- |
|  |  | No.scffold | match (bps) | ratio | No.scffold | match (bps) | ratio |
| chr1 | 122678785 | 530 | 118600142 | 96.68% | 426 | 118420406 | 96.53% |
| chr2 | 85426708 | 560 | 82326673 | 96.37% | 506 | 81325652 | 95.20% |
| chr3 | 91889043 | 491 | 88835371 | 96.68% | 386 | 88922239 | 96.77% |
| chr4 | 88276631 | 325 | 85763929 | 97.15% | 312 | 85888155 | 97.29% |
| chr5 | 88915250 | 388 | 86441518 | 97.22% | 311 | 86244417 | 97.00% |
| chr6 | 77573801 | 358 | 75024264 | 96.71% | 395 | 74724472 | 96.33% |
| chr7 | 80974532 | 344 | 78646082 | 97.12% | 306 | 78603442 | 97.07% |
| chr8 | 74330416 | 509 | 71467866 | 96.15% | 391 | 71362651 | 96.01% |
| chr9 | 61074082 | 361 | 58375518 | 95.58% | 330 | 57461507 | 94.08% |
| chr10 | 69331447 | 457 | 67257969 | 97.01% | 454 | 66665284 | 96.15% |
| chr11 | 74389097 | 385 | 71593524 | 96.24% | 395 | 71751951 | 96.45% |
| chr12 | 72498081 | 345 | 70269479 | 96.93% | 293 | 70291504 | 96.96% |
| chr13 | 63241923 | 284 | 61290104 | 96.91% | 360 | 60853763 | 96.22% |
| chr14 | 60966679 | 439 | 58287347 | 95.61% | 291 | 58946263 | 96.69% |
| chr15 | 64190966 | 400 | 61975391 | 96.55% | 348 | 61905327 | 96.44% |
| chr16 | 59632846 | 482 | 57365765 | 96.20% | 412 | 57441703 | 96.33% |
| chr17 | 64289059 | 335 | 62130659 | 96.64% | 319 | 62255205 | 96.84% |
| chr18 | 55844845 | 448 | 53749781 | 96.25% | 365 | 53416439 | 95.65% |
| chr19 | 53741614 | 278 | 51898542 | 96.57% | 264 | 52093591 | 96.93% |
| chr20 | 58134056 | 308 | 56490717 | 97.17% | 403 | 55702301 | 95.82% |
| chr21 | 50858623 | 345 | 48776874 | 95.91% | 246 | 49098143 | 96.54% |
| chr22 | 61439934 | 273 | 59511258 | 96.86% | 242 | 59738415 | 97.23% |
| chr23 | 52294480 | 261 | 50720913 | 96.99% | 231 | 50595715 | 96.75% |
| chr24 | 47698779 | 228 | 46229868 | 96.92% | 279 | 45615556 | 95.63% |
| chr25 | 51628933 | 314 | 49989618 | 96.82% | 274 | 49420535 | 95.72% |
| chr26 | 38964690 | 257 | 37321034 | 95.78% | 265 | 37366406 | 95.90% |
| chr27 | 45876710 | 308 | 44271466 | 96.50% | 251 | 44340250 | 96.65% |
| chr28 | 41182112 | 195 | 39860040 | 96.79% | 242 | 39325338 | 95.49% |
| chr29 | 41845238 | 245 | 40366346 | 96.47% | 205 | 40418993 | 96.59% |
| chr30 | 40214260 | 184 | 39002642 | 96.99% | 203 | 38869202 | 96.66% |
| chr31 | 39895921 | 207 | 38749138 | 97.13% | 291 | 38164748 | 95.66% |
| chr32 | 38810281 | 268 | 37264584 | 96.02% | 205 | 37533848 | 96.71% |
| chr33 | 31377067 | 141 | 30535365 | 97.32% | 106 | 30470653 | 97.11% |
| chr34 | 42124431 | 198 | 40839137 | 96.95% | 193 | 40830771 | 96.93% |
| chr35 | 26524999 | 149 | 25809790 | 97.30% | 143 | 25755402 | 97.10% |
| chr36 | 30810995 | 121 | 30159798 | 97.89% | 118 | 30109158 | 97.72% |
| chr37 | 30902991 | 133 | 30024350 | 97.16% | 137 | 29938874 | 96.88% |
| chr38 | 23914537 | 137 | 23198341 | 97.01% | 119 | 23067191 | 96.46% |
| chrX | 123869142 | 2418 | 106675548 | 86.12% | 844 | 111465783 | 89.99% |
| nonchr | 83326164 | 3228 | 14731239 | 17.68% | 3228 | 14933461 | 17.92% |
| sum | 2410960148 | 17637 | 2251827990 | 93.40% | 15089 | 2251334714 | 93.38% |

Supplementary Table 17. Genes located in the specific regions of the dog reference genome.

| protein_id | kegg_symbol | NCBI ID |
| --- | --- | --- |
| ENSCAFP00000036896 | cfa:487739 | 487739 |
| ENSCAFP00000014026 | cfa:478410 | 478410 |
| ENSCAFP00000038703 | cfa:478404 | 478404 |
| ENSCAFP00000028858 | cfa:491239 | 491239 |
| ENSCAFP00000035888 | cfa:611213 | 611213 |
| ENSCAFP00000043066 | cfa:609539 | 609539 |
| ENSCAFP00000035646 | cfa:609845 | 609845 |
| ENSCAFP00000039197 | cfa:608202 | 608202 |
| ENSCAFP00000040427 | cfa:491391 | 491391 |
| ENSCAFP00000041672 | cfa:608202 | 608202 |
| ENSCAFP00000042839 | cfa:609845 | 609845 |
| ENSCAFP00000043091 | cfa:607996 | 607996 |
| ENSCAFP00000035356 | cfa:609831 | 609831 |
| ENSCAFP00000036086 | cfa:609461 | 609461 |
| ENSCAFP00000039338 | pon:100442730 | 100442730 |
| ENSCAFP00000039040 | cfa:607314 | 607314 |
| ENSCAFP00000042844 | cfa:607460 | 607460 |
| ENSCAFP00000034594 | aml:100470731 | 100470731 |
| ENSCAFP00000012550 | cfa:482940 | 482940 |
| ENSCAFP00000034826 | cfa:609287 | 609287 |
| ENSCAFP00000034859 | cfa:491402 | 491402 |
| ENSCAFP00000042862 | cfa:607723 | 607723 |
| ENSCAFP00000033191 | cfa:608775 | 608775 |
| ENSCAFP00000034271 | cfa:610638 | 610638 |
| ENSCAFP00000043128 | cfa:607762 | 607762 |
| ENSCAFP00000009418 | aml:100466211 | 100466211 |
| ENSCAFP00000020076 | ecb:100056394 | 100056394 |
| ENSCAFP00000029205 | mmu:258543 | 258543 |
| ENSCAFP00000034827 | cfa:488680 | 488680 |
| ENSCAFP00000034963 | cfa:607362 | 607362 |
| ENSCAFP00000036224 | cfa:486189 | 486189 |
| ENSCAFP00000036460 | ecb:100056394 | 100056394 |
| ENSCAFP00000037963 | cfa:488680 | 488680 |
| ENSCAFP00000038783 | cfa:488676 | 488676 |
| ENSCAFP00000042802 | cfa:608013 | 608013 |
| ENSCAFP00000013368 | cfa:609830 | 609830 |
| ENSCAFP00000013070 | cfa:606887 | 606887 |
| ENSCAFP00000036362 | cfa:606887 | 606887 |
| ENSCAFP00000022618 | xtr:733891 | 733891 |
| ENSCAFP00000006448 | cfa:475531 | 475531 |
| ENSCAFP00000038647 | aml:100479417 | 100479417 |
| ENSCAFP00000043203 | aml:100475613 | 100475613 |
| ENSCAFP00000040757 | gga:416182 | 416182 |
| ENSCAFP00000039254 | cfa:608213 | 608213 |
| ENSCAFP00000038376 | cfa:611521 | 611521 |
| ENSCAFP00000037194 | mcc:716730 | 716730 |
| ENSCAFP00000030924 | ptr:748816 | 748816 |
| ENSCAFP00000033217 | ptr:748816 | 748816 |
| ENSCAFP00000005757 | cfa:610264 | 610264 |
| ENSCAFP00000042439 | cfa:608787 | 608787 |
| ENSCAFP00000029362 | cfa:607908 | 607908 |
| ENSCAFP00000036246 | cfa:608608 | 608608 |
| ENSCAFP00000039840 | cfa:607908 | 607908 |
| ENSCAFP00000041326 | cfa:607908 | 607908 |
| ENSCAFP00000043061 | cfa:608608 | 608608 |
| ENSCAFP00000031828 | cfa:612484 | 612484 |
| ENSCAFP00000040849 | cfa:612472 | 612472 |
| ENSCAFP00000034964 | cfa:609645 | 609645 |
| ENSCAFP00000040907 | cfa:608928 | 608928 |
| ENSCAFP00000013440 | ecb:100052430 | 100052430 |
| ENSCAFP00000033523 | cfa:607400 | 607400 |
| ENSCAFP00000040678 | cfa:607400 | 607400 |
| ENSCAFP00000024502 | bta:100337360 | 100337360 |
| ENSCAFP00000021049 | cfa:480772 | 480772 |
| ENSCAFP00000040742 | cfa:475610 | 475610 |
| ENSCAFP00000000477 | cfa:481143 | 481143 |
| ENSCAFP00000002564 | oaa:100076651 | 100076651 |
| ENSCAFP00000002766 | aml:100471480 | 100471480 |
| ENSCAFP00000005040 | bta:100337029 | 100337029 |
| ENSCAFP00000008097 | aml:100466538 | 100466538 |
| ENSCAFP00000009199 | cfa:609167 | 609167 |
| ENSCAFP00000009881 | aml:100482661 | 100482661 |
| ENSCAFP00000009894 | cfa:475937 | 475937 |
| ENSCAFP00000012074 | cfa:491531 | 491531 |
| ENSCAFP00000013331 | cfa:487737 | 487737 |
| ENSCAFP00000015708 | cfa:609691 | 609691 |
| ENSCAFP00000015957 | cfa:610301 | 610301 |
| ENSCAFP00000016194 | cfa:487799 | 487799 |
| ENSCAFP00000016280 | cfa:491711 | 491711 |
| ENSCAFP00000018542 | cfa:610328 | 610328 |
| ENSCAFP00000019979 | cfa:612180 | 612180 |
| ENSCAFP00000019984 | cfa:476770 | 476770 |
| ENSCAFP00000019992 | cfa:491364 | 491364 |
| ENSCAFP00000020278 | cfa:607401 | 607401 |
| ENSCAFP00000020349 | cfa:612181 | 612181 |
| ENSCAFP00000022486 | cfa:610478 | 610478 |
| ENSCAFP00000022548 | cfa:612177 | 612177 |
| ENSCAFP00000023351 | cfa:489070 | 489070 |
| ENSCAFP00000024222 | mdo:100012204 | 100012204 |
| ENSCAFP00000024868 | ssc:100154963 | 100154963 |
| ENSCAFP00000028529 | aml:100469103 | 100469103 |
| ENSCAFP00000028969 | cfa:612563 | 612563 |
| ENSCAFP00000031016 | cfa:491364 | 491364 |
| ENSCAFP00000031289 | cfa:480976 | 480976 |
| ENSCAFP00000031951 | aml:100477042 | 100477042 |
| ENSCAFP00000032067 | cfa:611804 | 611804 |
| ENSCAFP00000032310 | cfa:486374 | 486374 |
| ENSCAFP00000032684 | cfa:608365 | 608365 |
| ENSCAFP00000032992 | ssc:100154182 | 100154182 |
| ENSCAFP00000033046 | cfa:611136 | 611136 |
| ENSCAFP00000033054 | aml:100464942 | 100464942 |
| ENSCAFP00000033081 | ecb:100066211 | 100066211 |
| ENSCAFP00000033371 | cfa:612656 | 612656 |
| ENSCAFP00000033535 | cfa:475706 | 475706 |
| ENSCAFP00000033748 | cfa:482538 | 482538 |
| ENSCAFP00000033774 | cfa:491389 | 491389 |
| ENSCAFP00000033776 | cfa:485243 | 485243 |
| ENSCAFP00000033777 | cfa:477042 | 477042 |
| ENSCAFP00000033800 | cfa:609777 | 609777 |
| ENSCAFP00000033829 | cfa:403631 | 403631 |
| ENSCAFP00000034039 | cfa:608285 | 608285 |
| ENSCAFP00000034080 | cfa:477031 | 477031 |
| ENSCAFP00000034358 | cfa:609857 | 609857 |
| ENSCAFP00000034491 | cfa:608320 | 608320 |
| ENSCAFP00000034523 | cfa:609676 | 609676 |
| ENSCAFP00000034536 | cfa:609924 | 609924 |
| ENSCAFP00000035278 | cfa:606825 | 606825 |
| ENSCAFP00000035331 | cfa:609558 | 609558 |
| ENSCAFP00000035675 | cfa:491573 | 491573 |
| ENSCAFP00000035788 | cfa:610760 | 610760 |
| ENSCAFP00000035795 | cfa:480794 | 480794 |
| ENSCAFP00000035832 | mcc:694675 | 694675 |
| ENSCAFP00000035836 | cfa:480791 | 480791 |
| ENSCAFP00000035865 | cfa:609747 | 609747 |
| ENSCAFP00000035993 | cfa:612244 | 612244 |
| ENSCAFP00000036039 | cfa:609491 | 609491 |
| ENSCAFP00000036133 | cfa:491529 | 491529 |
| ENSCAFP00000036237 | cfa:607919 | 607919 |
| ENSCAFP00000036430 | cfa:478990 | 478990 |
| ENSCAFP00000036456 | cfa:608774 | 608774 |
| ENSCAFP00000036458 | cfa:486202 | 486202 |
| ENSCAFP00000036539 | mcc:708283 | 708283 |
| ENSCAFP00000036552 | cfa:607919 | 607919 |
| ENSCAFP00000036561 | cfa:608804 | 608804 |
| ENSCAFP00000036876 | cfa:609618 | 609618 |
| ENSCAFP00000036972 | ecb:100072447 | 100072447 |
| ENSCAFP00000037034 | bta:781039 | 781039 |
| ENSCAFP00000037086 | xla:443628 | 443628 |
| ENSCAFP00000037119 | cfa:491532 | 491532 |
| ENSCAFP00000037284 | cfa:477933 | 477933 |
| ENSCAFP00000037355 | aml:100470731 | 100470731 |
| ENSCAFP00000037392 | gga:769550 | 769550 |
| ENSCAFP00000037421 | aml:100468306 | 100468306 |
| ENSCAFP00000037434 | cfa:491389 | 491389 |
| ENSCAFP00000037720 | cfa:491436 | 491436 |
| ENSCAFP00000037724 | cfa:483579 | 483579 |
| ENSCAFP00000037728 | cfa:609179 | 609179 |
| ENSCAFP00000037729 | cfa:609558 | 609558 |
| ENSCAFP00000037878 | hsa:100506052 | 100506052 |
| ENSCAFP00000037988 | cfa:609657 | 609657 |
| ENSCAFP00000037997 | aml:100467714 | 100467714 |
| ENSCAFP00000038167 | cfa:475531 | 475531 |
| ENSCAFP00000038254 | cfa:487079 | 487079 |
| ENSCAFP00000038290 | cfa:475851 | 475851 |
| ENSCAFP00000038327 | cfa:609821 | 609821 |
| ENSCAFP00000038364 | cfa:608918 | 608918 |
| ENSCAFP00000038400 | cfa:482206 | 482206 |
| ENSCAFP00000038404 | aml:100477959 | 100477959 |
| ENSCAFP00000038455 | cfa:608804 | 608804 |
| ENSCAFP00000038499 | bta:100140226 | 100140226 |
| ENSCAFP00000038534 | cfa:477430 | 477430 |
| ENSCAFP00000038616 | cfa:442984 | 442984 |
| ENSCAFP00000038921 | cfa:612054 | 612054 |
| ENSCAFP00000039043 | cfa:612348 | 612348 |
| ENSCAFP00000039113 | cfa:609491 | 609491 |
| ENSCAFP00000039178 | cfa:480794 | 480794 |
| ENSCAFP00000039288 | cfa:492199 | 492199 |
| ENSCAFP00000039295 | cfa:482941 | 482941 |
| ENSCAFP00000039454 | rno:289990 | 289990 |
| ENSCAFP00000039539 | cfa:608873 | 608873 |
| ENSCAFP00000039550 | cfa:482941 | 482941 |
| ENSCAFP00000039584 | cfa:476574 | 476574 |
| ENSCAFP00000039611 | cfa:611533 | 611533 |
| ENSCAFP00000039646 | cfa:608032 | 608032 |
| ENSCAFP00000039675 | xla:443628 | 443628 |
| ENSCAFP00000039721 | cfa:607089 | 607089 |
| ENSCAFP00000039883 | cfa:612966 | 612966 |
| ENSCAFP00000039962 | cfa:610832 | 610832 |
| ENSCAFP00000040097 | cfa:483437 | 483437 |
| ENSCAFP00000040144 | cfa:612180 | 612180 |
| ENSCAFP00000040194 | ecb:100071004 | 100071004 |
| ENSCAFP00000040293 | cfa:610430 | 610430 |
| ENSCAFP00000040419 | cfa:609857 | 609857 |
| ENSCAFP00000040494 | cfa:477937 | 477937 |
| ENSCAFP00000040499 | cfa:608804 | 608804 |
| ENSCAFP00000040541 | cfa:609179 | 609179 |
| ENSCAFP00000040574 | cfa:612274 | 612274 |
| ENSCAFP00000040586 | cfa:476898 | 476898 |
| ENSCAFP00000040827 | cfa:607276 | 607276 |
| ENSCAFP00000041126 | cfa:477042 | 477042 |
| ENSCAFP00000041156 | cfa:480491 | 480491 |
| ENSCAFP00000041348 | cfa:483581 | 483581 |
| ENSCAFP00000041554 | cfa:609094 | 609094 |
| ENSCAFP00000041564 | cfa:484145 | 484145 |
| ENSCAFP00000041583 | xla:443628 | 443628 |
| ENSCAFP00000041599 | ecb:100034010 | 100034010 |
| ENSCAFP00000041622 | cfa:483579 | 483579 |
| ENSCAFP00000041655 | cfa:492209 | 492209 |
| ENSCAFP00000041699 | cfa:608656 | 608656 |
| ENSCAFP00000041760 | cqu:CpipJ_CPIJ012420 | CpipJ_CPIJ012420 |
| ENSCAFP00000042036 | cfa:610430 | 610430 |
| ENSCAFP00000042107 | ecb:100054816 | 100054816 |
| ENSCAFP00000042122 | cfa:612050 | 612050 |
| ENSCAFP00000042199 | gga:426023 | 426023 |
| ENSCAFP00000042286 | cfa:612762 | 612762 |
| ENSCAFP00000042573 | bta:100336997 | 100336997 |
| ENSCAFP00000042624 | cfa:610897 | 610897 |
| ENSCAFP00000042660 | cfa:479385 | 479385 |
| ENSCAFP00000042742 | cfa:610053 | 610053 |
| ENSCAFP00000042873 | ecb:100064590 | 100064590 |
| ENSCAFP00000042942 | ecb:100053695 | 100053695 |
| ENSCAFP00000042959 | cfa:609179 | 609179 |
| ENSCAFP00000043108 | cfa:612408 | 612408 |
| ENSCAFP00000043111 | rno:257642 | 257642 |
| ENSCAFP00000043196 | cfa:403691 | 403691 |

Supplementary Table 18. lincRNAs located in the specific regions of the dog reference genome.

| **Transcript ID** | **Gene ID** |
| --- | --- |
| CFRNASEQ_IGNC_Spliced_00031687_1 | RLOC_00021429 |
| CFRNASEQ_IGNC_Spliced_00031691_1 | RLOC_00021432 |
| TCONS_00042498 | RLOC_00007818 |
| CFRNASEQ_IGNC_Spliced_00052181_1 | RLOC_00018287 |
| CFRNASEQ_IGNC_Spliced_00063610_1 | RLOC_00008547 |
| CFRNASEQ_IGNC_Spliced_00059949_1 | RLOC_00008554 |
| CFRNASEQ_IGNC_Spliced_00160435_1 | RLOC_00029983 |
| TCONS_00182035 | RLOC_00026243 |
| TCONS_00198805 | RLOC_00011884 |
| CFRNASEQ_IGNC_Spliced_00178258_1 | RLOC_00011894 |
| CFRNASEQ_IGNC_Spliced_00178259_1 | RLOC_00011895 |
| CFRNASEQ_IGNC_Spliced_00178455_1 | RLOC_00011968 |
| CFRNASEQ_IGNC_Spliced_00185230_1 | RLOC_00036165 |
| CFRNASEQ_IGNC_Spliced_00185394_1 | RLOC_00036115 |
| CFRNASEQ_IGNC_Spliced_00185396_1 | RLOC_00035949 |
| TCONS_00203260 | RLOC_00036222 |
| TCONS_00203262 | RLOC_00036223 |
| CFRNASEQ_IGNC_Spliced_00185553_1 | RLOC_00036200 |
| CFRNASEQ_IGNC_Spliced_00185660_1 | RLOC_00031856 |
| CFRNASEQ_IGNC_Spliced_00185674_1 | RLOC_00031862 |
| CFRNASEQ_IGNC_Spliced_00185853_1 | RLOC_00035840 |
| CFRNASEQ_IGNC_Spliced_00186061_1 | RLOC_00035796 |
| CFRNASEQ_IGNC_Spliced_00186311_1 | RLOC_00035955 |
| TCONS_00204073 | RLOC_00036220 |
| TCONS_00204157 | RLOC_00036225 |
| CFRNASEQ_IGNC_Spliced_00186490_1 | RLOC_00036073 |
| CFRNASEQ_IGNC_Spliced_00186491_1 | RLOC_00036073 |
| CFRNASEQ_IGNC_Spliced_00186723_1 | RLOC_00036118 |
| TCONS_00204445 | RLOC_00036227 |
| CFRNASEQ_IGNC_Spliced_00186885_1 | RLOC_00036097 |
| TCONS_00204511 | RLOC_00036226 |

Supplementary Table 19. GO and KEGG enrichment analysis of dog-specific genes.

| Category | Term | Genes | P-Value |
| --- | --- | --- | --- |
| KEGG_PATHWAY | Olfactory transduction | 13 | 2.30E-05 |
| KEGG_PATHWAY | Ribosome | 6 | 9.50E-04 |
| KEGG_PATHWAY | Porphyrin and chlorophyll metabolism | 3 | 1.50E-02 |
| KEGG_PATHWAY | Drug metabolism | 3 | 1.90E-02 |
| KEGG_PATHWAY | Starch and sucrose metabolism | 3 | 2.40E-02 |
| KEGG_PATHWAY | Metabolism of xenobiotics by cytochrome P450 | 3 | 2.80E-02 |

Supplementary table 20. The functional impact of SVs in the dog-dhole genomic alignment.

| **SV** | **sum** | **Cds** | **exon** | **intron** | **UTR** | **intergenic region** |
| --- | --- | --- | --- | --- | --- | --- |
| **inversion** | 463 | 22 | 26 | 91 | 7 | 314 |
| **Repeat** | 4248 | 555 | 670 | 712 | 154 | 2952 |
| **insertion** | 115937 | 1054 | 2338 | 24317 | 1438 | 74508 |
| **deletion** | 63809 | 569 | 1146 | 13030 | 431 | 41691 |
| **Translocation** | 1416 | 256 | 290 | 619 | 80 | 400 |

Supplementary table 21. The functional impact of SVs in Dog-wolf genomic alignment.

| **SV** | **sum** | **Cds** | **Exon** | **intron** | **UTR** | **intergenic region** |
| --- | --- | --- | --- | --- | --- | --- |
| **inversion** | 368 | 46 | 49 | 72 | 11 | 239 |
| **Repeat** | 3921 | 541 | 630 | 712 | 143 | 2727 |
| **insertion** | 76889 | 5037 | 6556 | 16516 | 3071 | 48311 |
| **deletion** | 28482 | 1431 | 1819 | 5496 | 632 | 18846 |
| **Translocation** | 1618 | 490 | 541 | 769 | 120 | 392 |

Supplementary table 22. The functional impact of SVs in the multiz alignment of three Canina genomes.

| **SV** | **Multiz** | **cds** | **exon** | **Intron** | **UTR** | **intergenic region** |
| --- | --- | --- | --- | --- | --- | --- |
| **inversion** | 15 | 0 | 0 | 3 | 0 | 12 |
| **Repeat** | 443 | 47 | 55 | 121 | 14 | 307 |
| **insertion** | 16315 | 89 | 231 | 5204 | 122 | 10641 |
| **deletion** | 2565 | 31 | 56 | 757 | 26 | 1725 |
| **Translocation** | 32 | 4 | 6 | 8 | 0 | 16 |

Supplementary Table 23. The summary of Transcriptome of 5 indigenous dogs from China.

|  | dog01 | dog02 | dog03 | dog04 | dog05 |
| --- | --- | --- | --- | --- | --- |
| Raw data (Gb) | 4.376 | 5.442 | 9.276 | 5.633 | 7.842 |
| Total mapped pair | 16307040 | 20745738 | 34756876 | 18680049 | 26318920 |
| Mapping rate(%) | 89.4 | 90.1 | 87.8 | 86.7 | 86.7 |
